# Characterizing the Effects of Adding Virtual and Augmented Reality in Robot-Assisted Training

**DOI:** 10.1109/TNSRE.2024.3432661

**Published:** 2024-07-31

**Authors:** Xupeng Ai, Victor Santamaria, Sunil K. Agrawal

**Affiliations:** Department of Mechanical Engineering, Columbia University, New York, NY 10027 USA; Department of Mechanical Engineering, Columbia University, New York, NY 10027 USA; Rehabilitation Sciences Department, Physical Therapy Division, New York Medical College, Valhalla, NY 10595 USA; Department of Mechanical Engineering and the Department of Rehabilitation and Regenerative Medicine, Columbia University, New York, NY 10027 USA

**Keywords:** Augmented reality, extended reality, posture training, robot-assisted training, virtual reality

## Abstract

Extended reality (XR) technology combines physical reality with computer synthetic virtuality to deliver immersive experience to users. Virtual reality (VR) and augmented reality (AR) are two subdomains within XR with different immersion levels. Both of these have the potential to be combined with robot-assisted training protocols to maximize postural control improvement. In this study, we conducted a randomized control experiment with sixty-three healthy subjects to compare the effectiveness of robot-assisted posture training combined with VR or AR against robotic training alone. A robotic Trunk Support Trainer (TruST) was employed to deliver assistive force at the trunk as subjects moved beyond the stability limits during training. Our results showed that both VR and AR significantly enhanced the training outcomes of the TruST intervention. However, the VR group experienced higher simulator sickness compared to the AR group, suggesting that AR is better suited for sitting posture training in conjunction with TruST intervention. Our findings highlight the added value of XR to robot-assisted training and provide novel insights into the differences between AR and VR when integrated into a robotic training protocol. In addition, we developed a custom XR application that suited well for TruST intervention requirements. Our approach can be extended to other studies to develop novel XR-enhanced robotic training platforms.

## I. Introduction

SPATIAL control of body segments across postures and contexts demands simultaneous control of postural orientation and stability. This ability develops through complex cognitive, sensory, and motor experiences [1]. It involves integrating sensory inputs from visual, somatosensory, and vestibular systems to generate adaptive postural responses through the nervous and musculoskeletal systems [2]. Postural control is essential for people of all ages and abilities, as it directly impacts daily activities such as grooming, dressing, and eating. However, this ability may be impaired in people with moderate-to-severe neuromotor deficits such as in stroke, spinal cord injury (SCI), and cerebral palsy (CP), leading to challenges in daily living [3].

In rehabilitation, conventional training (CT) strategies, such as core muscle strengthening, joint locking, hippotherapy, and proprioceptive training, are known to improve sitting postural control [4]. For example, Moraes et al. conducted a longitudinal study to investigate the sitting balance performance of 13 CP children after 12, 24, and 36 hippotherapy sessions and at a 45-day follow-up [5]. Their findings indicate that continued CT could contribute to sustained enhancements in seated postural balance, emphasizing the potential for long-term benefits of CT and the necessity of ongoing treatment. However, providing sufficiently rich intervention in CT can be labor-intensive and expensive [6], [7]. Additionally, simple repetitive movements involved in CT may not be stimulating for patients, leading to a lack of enthusiasm to continue the treatments [8], [9]. Therefore, researchers are exploring novel rehabilitation technologies to overcome these limitations.

Robot-assisted training (RAT) is an emerging technology applied in the recovery of postural control deficits. It offers several advantages over CT, such as standardized training environments, adaptable support, increased intervention intensity, and reduced physical burden on therapists [10]. In our previous work, we have developed a cable-driven robotic rehabilitation platform, Trunk Support Trainer (TruST), that can apply force to the trunk [11]. During training, subjects practice multidirectional reach tasks while receiving assistive forces at the trunk as they move their trunk beyond the stability limits. In a longitudinal study involving four children with CP [12], we found that after 12 TruST-intervention sessions, there was a significant expansion of sitting workspace, improved functional reach, and enhanced gross motor function. These improvements were well-maintained at the three-month follow-up.

**Fig. 1.**
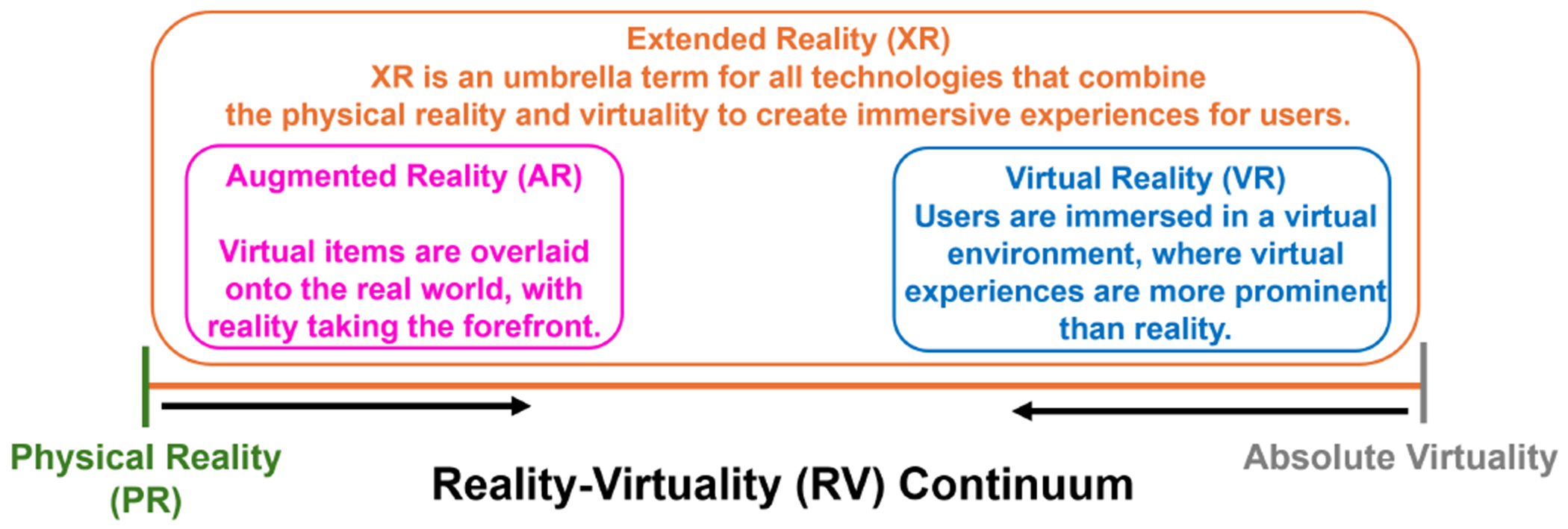
Definitions of extended reality (XR), augmented reality (AR), and virtual reality (VR). The Reality-Virtuality (RV) continuum represents the immersion level between absolute virtuality and physical reality. It encompasses all variations and compositions of real and virtual objects. The area between two extremes is XR, which includes both AR and VR.

Extended reality (XR) is also gaining popularity in posture training [13], [14]. XR serves as an umbrella term encompassing all technologies that blend physical reality with synthetic virtuality to create immersive interactive experiences for users [15]. Within the XR domain, two primary subgroups exist: virtual reality (VR) immerses users in 3D-rendered virtual environments, emphasizing virtual experiences; augmented reality (AR) superimposes virtual items onto the real world, with the physical real environment taking the forefront. To clarify the relationship between XR, VR, and AR, Milgram et al. introduced the concept of a reality-virtuality (RV) continuum [16]. As shown in Fig. 1, this continuum reflects the combination level of reality and virtuality, also known as immersion level. VR and AR sit close to the right end (absolute virtuality [17]) and left end (physical reality), respectively. Both VR and AR fall under the broader category of XR, which spans the entire continuum.

Extended reality (XR) has demonstrated effectiveness in enhancing motor performance, triggering neurophysiological changes, and stimulating neural plasticity [18], [19], [20]. In a study by Hesam-Shariati et al. [21], tri-axial accelerometers were used to capture movement in the more affected arm of chronic stroke patients. They assessed the motor control level of 24 patients during a 14-day XR therapy protocol, with a 6-month follow-up. Their findings suggest that continued XR therapy leads to long-lasting improvements in functional movement performance and upper-limb flexibility. Posture rehabilitation is a multi-disciplinary and multi-modal endeavor. To further improve motor recovery, a recent research trend involves incorporating XR into RAT. For instance, Manuli et al. [22] and Calabrò et al. [23], [24] applied VR to the Lokomat robotic platform, resulting in more significant improvements in cognitive recovery and motor control compared to CT.

Although the combined XR-RAT method has shown promising results in posture training, the additive effect of XR to RAT remains unclear and can be further investigated [25], [26]. Existing literature on combined XR-RAT methods predominantly employs CT as the control group, with limited comparisons to a RAT control group [27]. Consequently, it remains challenging to ascertain whether the observed benefits are attributable to the addition of XR or solely due to RAT itself. Furthermore, prior studies typically focused on a single XR subcategory–either AR or VR [27]. Given the distinct immersion levels of AR and VR (see Fig. 1), it is essential to investigate which modality (VR or AR) is more suitable for integration with RAT. Therefore, the purpose of this paper is to conduct a comparative study with a RAT control group (using the TruST robot) to assess the added value of XR to RAT and investigating differences between VR and AR when combined with RAT. To achieve this, we have formulated two research questions for this study.

Our primary question is whether introducing XR into TruST intervention could produce significantly different training outcomes from TruST intervention alone. During training, TruST provides assistive forces to help maintain balance when subjects move beyond their sitting stability limits [11]. The gamification feature of XR may motivate subjects to use the assistive force from TruST more proactively to improve postural control strategies. Therefore, we hypothesize that integrating XR into TruST intervention could lead to significant improvements in the training outcomes. Our secondary question is whether VR or AR is more effective when combined with the TruST intervention. Compared to AR, the higher immersion level in VR may pose a greater risk of simulator sickness [28]. The discomfort could be exacerbated by frequent and rapid head movements that are typical in sitting posture training. Additionally, numerous virtual objects presented in VR experience could cause cognitive overload and distract patients from RAT tasks [27], [29], [30]. Taking these factors into account, we hypothesize that AR performs better than VR when integrated with the TruST intervention.

## II. Methodology

### A. Subjects

Sixty-three healthy subjects participated in this study (age = 17–51; females = 27; left-handed = 4; mean height = 171.3 ± SE = 5.1 cm and mean weight = 67.4 ± SE = 10.4 kg). Approval for all ethical and experimental procedures in this paper was sought and granted by the Institutional Review Board (IRB) of Columbia University under Protocol No. AAAQ7781. Informed consent was received from all human subjects. The IRB approval date was 10/11/2023.

### B. Robotic Platform and XR Devices

**Fig. 2.**
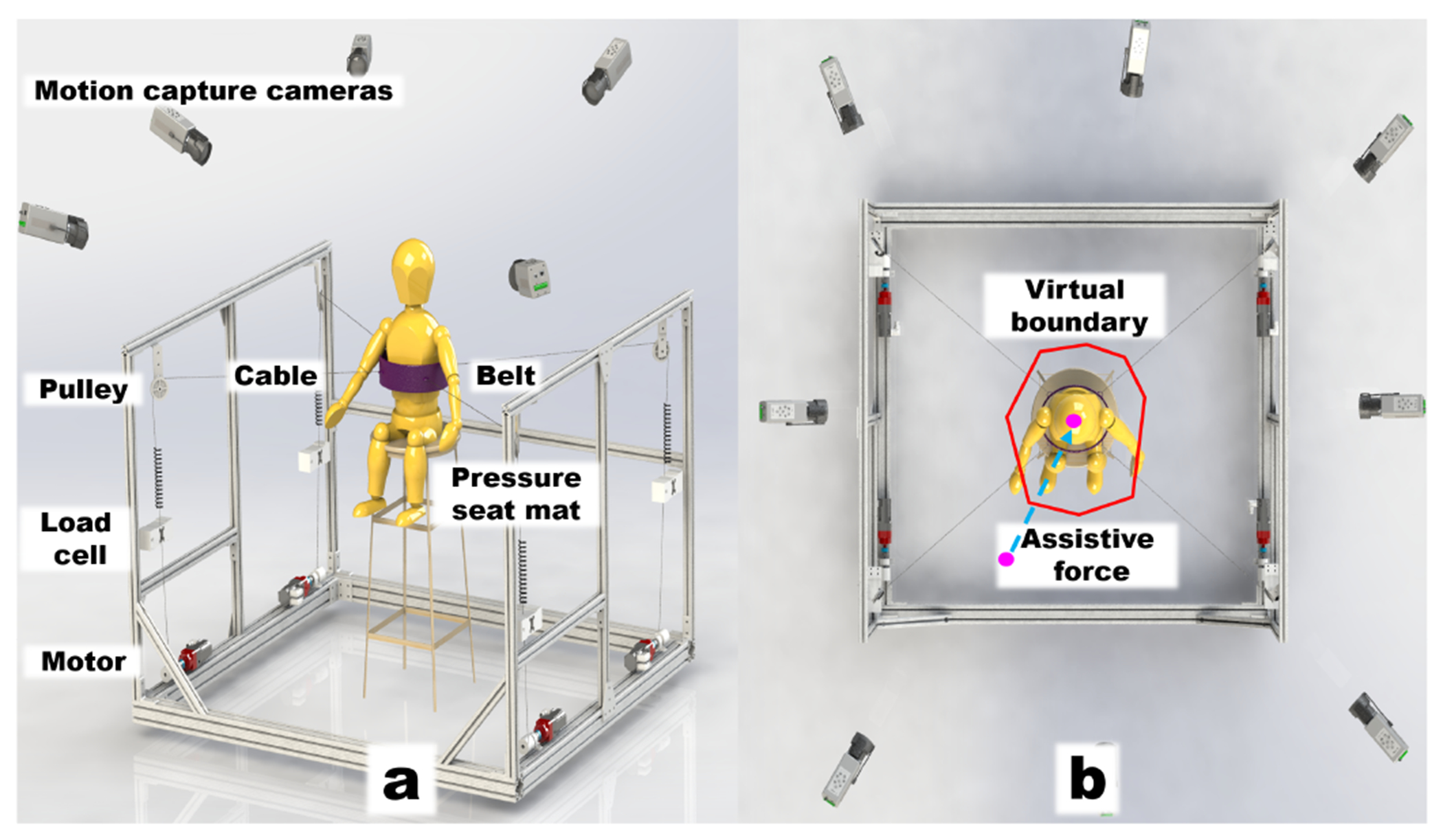
**a.** Schematic of the Trunk Support Trainer (TruST). **b.** Schematic of the assist-as-needed force field. During training, when the trunk moves beyond seated postural limits (represented by a star-shape virtual boundary), TruST generates a planar force towards the subject’s neutral position to help maintain balance.

As shown in Fig. 2a, TruST is a cable-driven RAT platform. Four cables are attached to a belt at the trunk. Four motors (Maxon Motor, Switzerland) instrumented with load-cells (LSB302 Futek, CA) are mounted on a stationary frame to control the cable tensions. Motion capture cameras (Vicon Vero 2.2, Denver) provide real-time position and orientation of the belt to the robotic controller. When the estimated trunk center (pink point in Fig. 2b) moves beyond the sitting stability boundary, TruST applies an assistive force (blue arrow in Fig. 2b) to help subjects maintain balance. Details of the TruST control mechanism are described in our previous work [11].

**Fig. 3.**
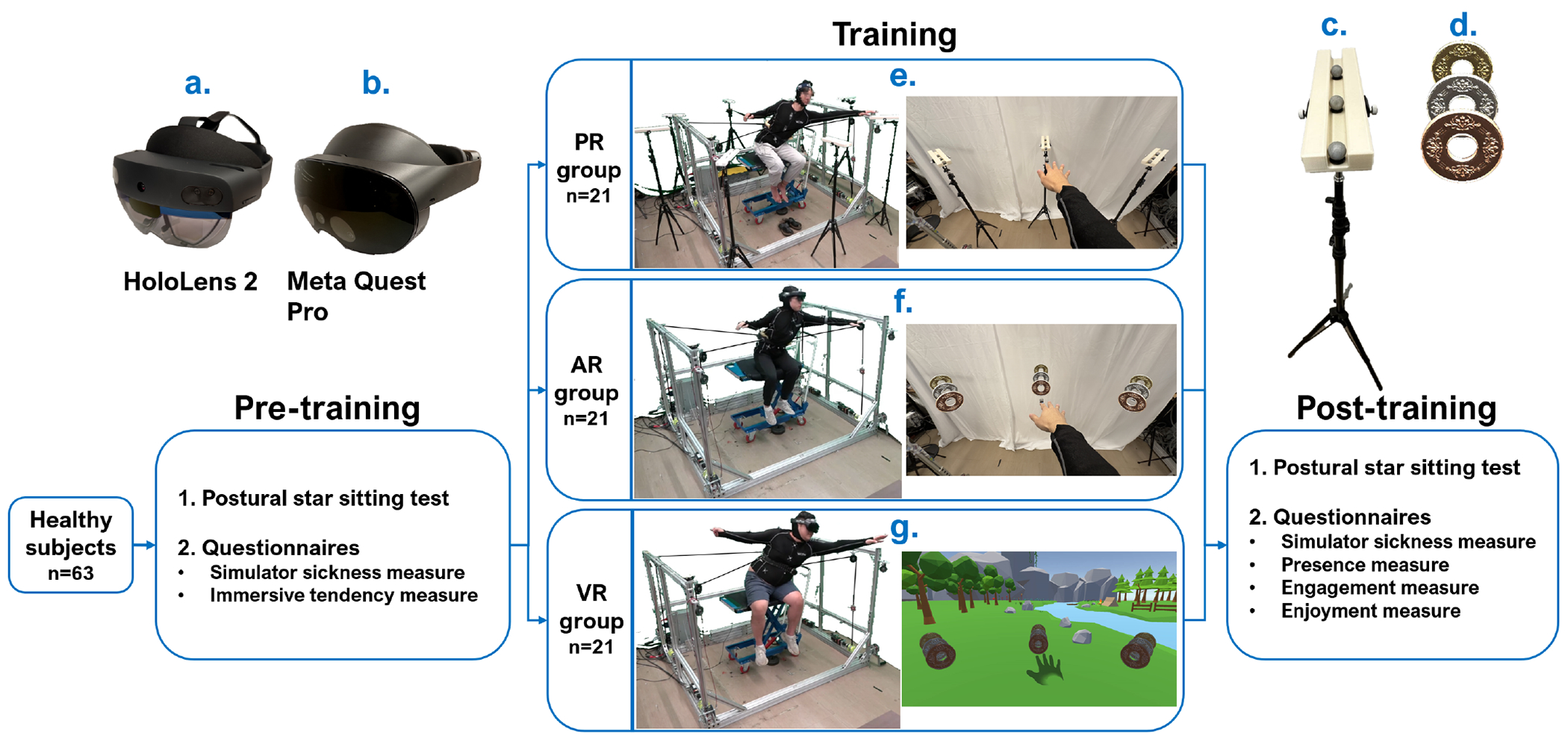
Schematic of the experiment setup **a.** HoloLens 2: used to deliver AR experience **b.** Meta Quest Pro: used to deliver VR experience **c.** Physical real reaching targets **d.** Virtual reaching targets **e, f, g.** The third-person and first-person views of training in PR, AR, VR groups, respectively.

Two flagship XR head mounted devices (HMDs) available on the market, Microsoft HoloLens 2 (Fig. 3a) and Meta Quest Pro (Fig. 3b), were used to deliver AR and VR experiences to subjects in this study, respectively. Both devices were determined to be usable, reliable, and effective in rehabilitation [31], [32]. Unity3D Engine (version 2021.3.21f1) and the MRTK3 package (Microsoft, WA) were used for the development of custom XR game application and cross-platform deployment. The application is available to the research community upon request consistent with the IRB guidelines (Unity project access link: https://roar.me.columbia.edu/content/trust).

### C. Experiment Setup

The schematic diagram of the study design is shown in Fig. 3. The experiment consists of three stages: pre-training test session, training session, and post-training test session.

Before training, a postural star sitting test (PSST) was performed [33]. Subjects wore a trunk belt and sat in the TruST without foot support. Cables were removed from the belt. Subjects were instructed to reach in eight principal directions. They used the dominant arm for the directions: front (F), front-dominant (FD), dominant (D), back-dominant (BD), and back (B); and the nondominant arm for the remaining three directions: front-nondominant (FND), nondominant (ND), and back-nondominant (BND). Before each reach, subjects sat upright and extended the arm to 90 degrees of shoulder flexion. Then, they were instructed to reach as far as possible without losing balance. The distance between their start and farthest reaching positions of the index fingertip was referred to as the functional reach test score (FRTS) in that direction [34]. Twenty-nine reflective markers were placed on anatomical landmarks to monitor their upper body movements and estimate the center of mass (COM). Marker attachment positions were selected based on the literature [35]. Nineteen VICON cameras (Fig. 2a) surrounding the subjects recorded marker trajectories at 100Hz. The center of pressure (COP) data were collected by a pressure seated mat (Tactilus, NY) at 56 Hz. Subjects were also asked to complete two standard questionnaires: Simulator Sickness Questionnaire (SSQ) [36] and Immersive Tendency Questionnaire (ITQ) [37].

The belt movement trajectory during the PSST was sent to the TruST controller to define the virtual boundary. Sixty-three subjects were randomly assigned to three equal groups: physical reality (PR) group, AR group, and VR group. Reaching targets were categorized into three difficulty levels: basic, medium, and hard. In the PR group, targets were represented as real reflective markers (Fig. 3c), while in the AR and VR groups, they were depicted as virtual bronze, silver, and gold coins (Fig. 3d). For all subjects, the basic level targets were placed at their farthest reaching positions in PSST, while the medium and hard level targets were placed at 10% and 20% FRTS farther away from the basic level targets, respectively.

During training, subjects were instructed to complete 96 bouts (12 rounds × 8 directions) of reach task with the assistance of TruST. The reaching direction sequence was shuffled across all rounds for all subjects. For each reach, basic, medium, and hard level targets accounted for scores 1, 2, and 3, respectively. Subjects were encouraged to score as high as possible. Fig. 3 e, f, g show the third-person and first-person views of the training session in PR, AR, and VR groups, respectively. Training environments were real in PR and AR groups but virtual in the VR group.

After training, subjects performed the PSST again and filled out four standard questionnaires: SSQ, Witmer & Singer Presence Questionnaire (PQ) [37], Game Engagement Questionnaire (GEQ) [38], and Universal Enjoyment Questionnaire (UEQ) [39].

### D. Data Preprocessing

MATLAB (Mathworks, MA) was used for data preprocessing. Data collected from VICON cameras and the pressure seated mat were lowpass filtered using a fourth-order Butterworth filter with cutoff frequencies of 10 and 6 Hz, respectively [40], [41]. For the self-report data collected from questionnaires, a seven-point Likert scale was assigned to each question [37]. We then normalized the total score of each answer sheet to the range 0–1 by dividing the sum of all question scores by the total possible score.

### E. Training Outcome Measures

#### 1) FRTS:

FRTS assesses the maximum distance a subject can reach while maintaining stability. We used it to evaluate upper limb function and sitting proactive balance for each direction in PSST. This measure has demonstrated high reliability and validity in existing literature [34].

#### 2) Sitting Workspace Area:

We extracted the upper-body COM trajectories in PSST and identified the farthest reach points for eight directions to define the sitting stability limits. Connecting these points formed a polygon that represents the sitting workspace. Its area serves as a measure of overall sitting dynamic balance ability [42] and functional independence [12].

#### 3) COP Variables:

Subjects sat on a pressure mat without foot support during PSST. Reaction forces exerted by the support surface converged at a single point, referred to as the COP. Total excursion and mean velocity are two widely used COP measures to assess how far and how quickly subjects shifted their COM within the base of support during multidirectional reaches [43]. Approximate entropy (ApEn) is a statistical metric used to quantify the regularity and predictability of time-sequential data [44]. It also serves as a standard COP measure in rehabilitation to assess the complexity of sitting posture control and flexibility in coordinating upper body segments [45]. ApEn values fall within the range of 0–2, with higher values indicating greater complexity.

#### 4) HIAR:

Active practice amount is critical for measuring training intensity and promoting neural plasticity [46]. To assess this factor, we first extracted the index fingertip trajectory during the baseline PSST. Next, we constructed a polygon that encompasses all trajectory points, representing the baseline reaching workspace. For each subject, we recorded both the total training time and the time spent outside the polygon (i.e., high intense activity time). The high intense activity rate (HIAR) is defined in Equation (1). We used this metric to assess the active practice time in exploring beyond baseline reach limits with the assistance of TruST.

(1) $$\text{HIAR}=\frac{\text{high intense activity time}}{\text{total training time}}$$

#### 5) Reach Task Score:

Challenging goal-oriented repetitions could foster motor skill exploration in posture training [46]. In this study, the basic, medium, and hard level reaching targets accounted for scores 1, 2, and 3, respectively. For each subject, we calculated the cumulative score for 96 reaches during training to assess motor task performance. The reach task score is another metric to measure the training intensity.

#### 6) SSQ Score:

Simulator sickness describes the phenomenon that subjects feel uncomfortable interacting with simulated environments. This discomfort arises when visual cues suggest self-motion, but the vestibular system does not detect the corresponding inertial forces [36]. Simulator sickness usually manifests as nausea, vomiting, eye fatigue, dizziness, ataxia, etc. These symptoms can be exacerbated during XR experiences and RAT with limited training space [28]. In this study, we used SSQ as a self-report symptom checklist to assess the severity of 16 symptoms related to simulator sickness in subjects before and after training.

#### 7) Immersion Score:

Immersion level refers to the capacity of an XR system to provide simulated virtual experiences to users. It is an objective metric influenced by both hardware properties (e.g., field of view, rendering frame rate) and software design (e.g., textures, brightness, and interaction design). In this study, we adopted the method proposed by Selzer and Castro [47] to calculate immersion scores for our AR and VR systems. These scores were then normalized to the range 0–1, representing the relative positions of our AR and VR systems on the RV continuum (PR group defaulted to 0).

#### 8) ITQ Score:

Immersive tendency refers to the subjective inclination to become deeply involved in situations and maintain focus on current activities. People with high immersive tendencies may easily ignore external distractions and fully engage in XR experiences, often becoming unaware of their immediate environment and the passage of time [37]. In this study, we used ITQ score to assess the immersive tendency ability of each subject before training. An example question from the ITQ is “Do you ever become so involved in movies, TV dramas, or books that you are not aware of things happening around you?”

#### 9) PQ Score:

Presence refers to the feeling of being physically present in a virtual environment and perceiving oneself as part of the digital world. It is a subjective metric influenced by psychological state, emotional fluctuation, and XR intervention quality [37]. We used PQ score to assess the presence level during training in the VR and AR groups (PR group defaulted to zero). An example question from the PQ is “How compelling was your sense of moving around inside the virtual environment?”

#### 10) GEQ Score:

Engagement refers to the involvement and attention that subjects exhibit when playing a game. It is a subjective metric influenced by mind flow state, psychological absorption, and dissociation [38]. We use the GEQ score to assess the engagement level of each subject during training. An example question from GEQ is “Do you feel that time seems to kind of stand still or stop during training?”

#### 11) UEQ Score:

Enjoyment refers to positive experience and satisfaction when playing a game. It is a subjective construct influenced by items such as pleasure, competence, and task challenge level [39]. We used UEQ score to assess the enjoyment level of each subject when performing the training task. An example question from UEQ is: “Do you feel that the activity was pleasurable to you?”

### F. Statistical Analysis

We conducted statistical analysis using SPSS (IBM, v29). The significance level was set at 0.05. We employed the Shapiro-Wilk test and visually inspected Q-Q plots to assess data normality.

Mixed between-within group analysis of variance (ANOVA) were performed to assess the impact of the different interventions (PR, AR, and AR groups) on several variables: FRTS, sitting workspace area, COP variables, and simulator sickness level across two time periods (pre-training and post-training). We examined homoscedasticity using Levene’s tests, respectively. If the ANOVA model was significant, we performed post-hoc test with Bonferroni’s inequality procedure for multiple comparisons.

One-way between-groups ANOVA were conducted to compare various measures (HIAR, reach task score, ITQ score, PQ score, GEQ score, and UEQ score) between the three groups. If the ANOVA model indicated significance, we followed up with post-hoc tests using Bonferroni’s inequality procedure.

## III. Results

### A. Immersion and Presence

**Fig. 4.**
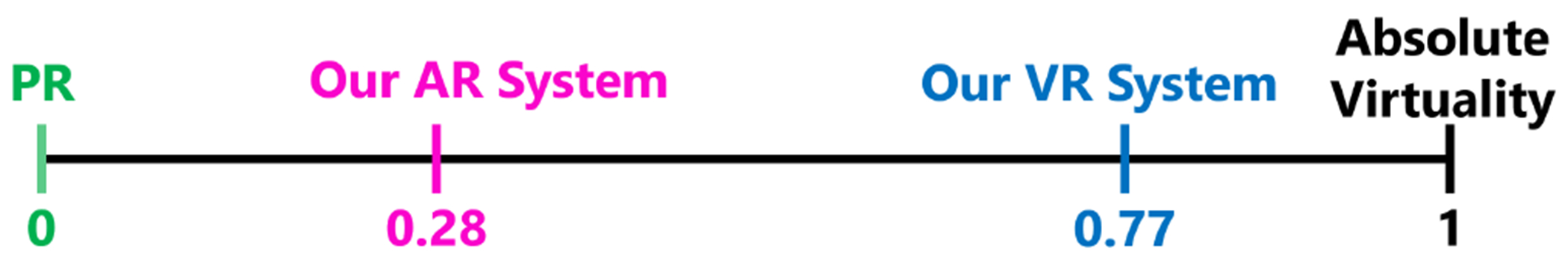
Normalized immersion scores for our AR and VR systems. PR group defaulted to 0.

Fig. 4 shows the normalized immersion scores for the AR and VR systems developed in this study. AR and VR groups sit close to the left and right ends of the spectrum, which follows the general trend in the RV continuum (Fig. 1).

No significant differences (*F*(2, 60) = 0.41, *p* = 0.67, *η*^2^ = 0.01) were found in ITQ scores (mean ± SD) among the PR group (0.50±0.06), the AR group (0.51±0.06), and the VR group (0.49 ± 0.05). Subjects in three groups showed similar immersive tendency before training.

After training, there was a statistically significant difference in the PQ scores reported by the AR group (0.38 ± 0.03) and the VR group (0.67 ± 0.05): *F*(1, 40) = 448.64, *p* < 0.001, *η*^2^ = 0.92. Subjects in the VR group felt a higher presence than those in the AR group during training.

### B. Functional Reach Performance and Balance

**Fig. 5.**
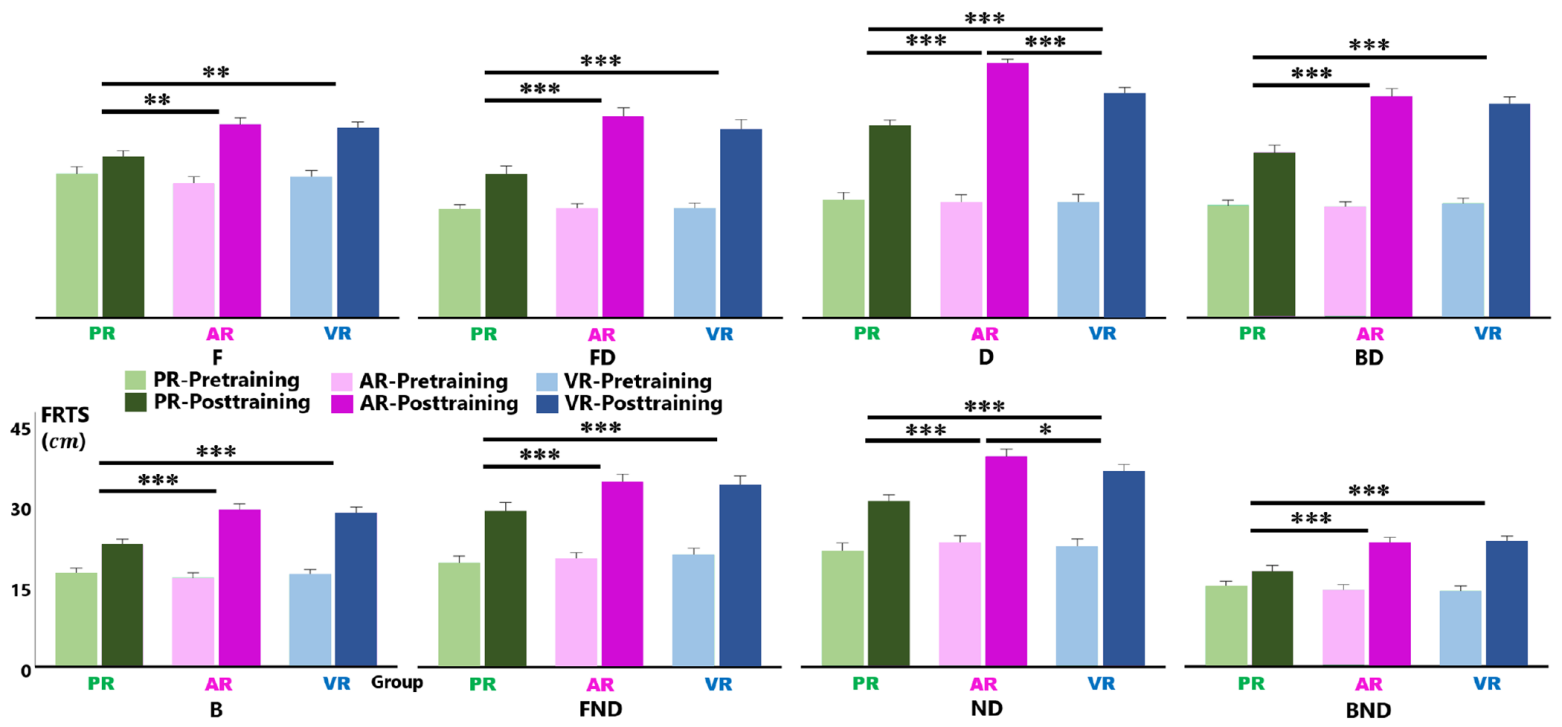
Baseline and post-training average FRTSs of PR, AR, and VR groups in eight reaching directions: front (F), front-dominant (FD), dominant (D), back-dominant (BD), and back (B), front-nondominant (FND), nondominant (ND), and back-nondominant (BND). Error bar = 95% CI. Significant pairwise differences between groups are denoted by black bars and asterisks. * *p* < 0.05, ** *p* < 0.01, *** *p* < 0.001.

The average FRTS for each group was visually depicted in Fig. 5. After training, all groups exhibited a significant increase of FRTS (*p* < 0.001) in each direction. Across all directions, the interaction effect between intervention group and test time was statistically significant (*p* < 0.01). As shown in Fig. 5, the increase was significantly greater in the AR and VR groups compared to the PR group. However, there was no significant difference in FRTS improvement between the AR and VR groups, except for the dominant and nondominant sides (see Fig. 5 D, ND).

**Fig. 6.**
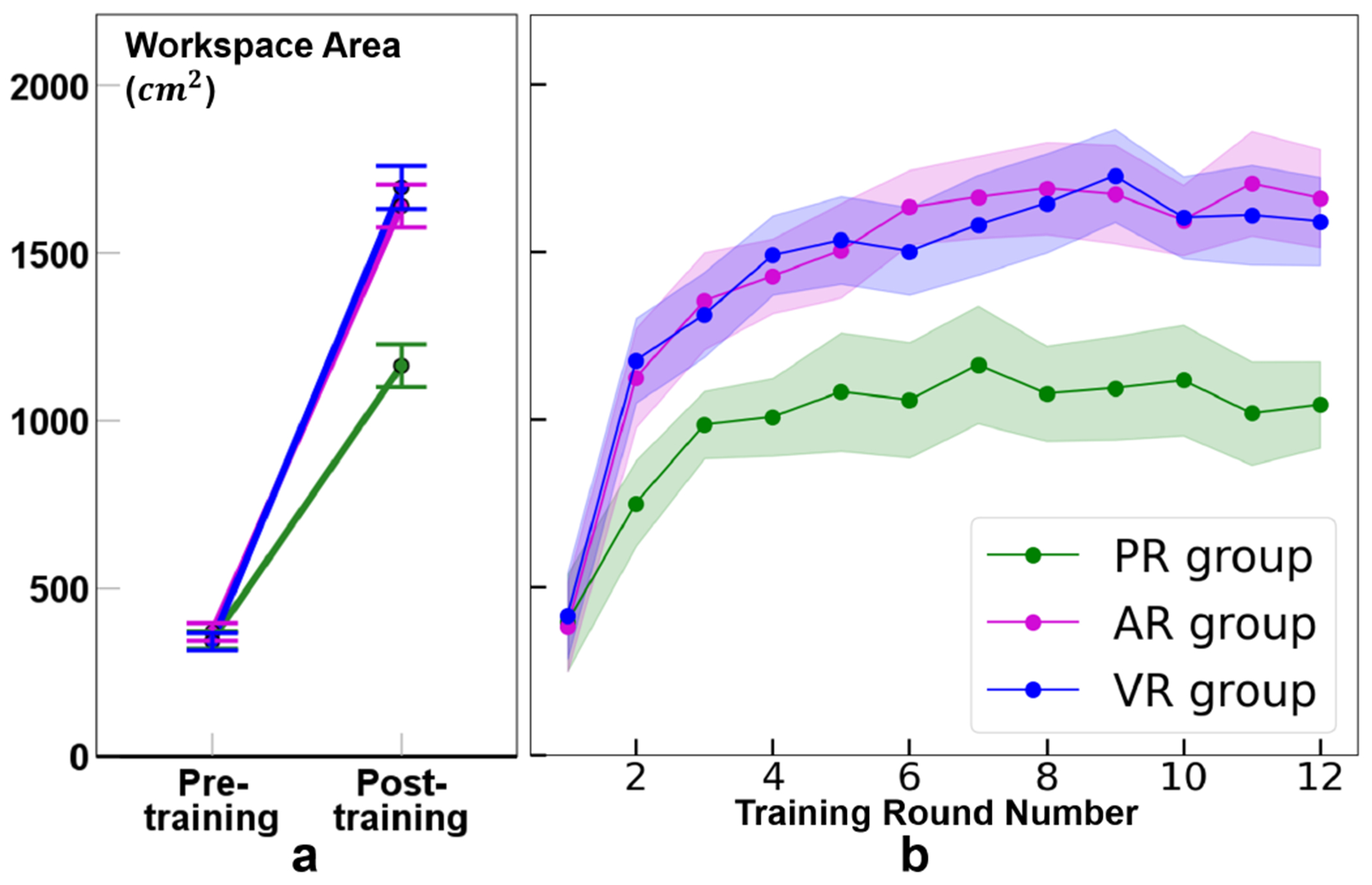
**a.** Average sitting workspace areas for PR, AR, and VR groups in the pre- and post-training tests. Error bar = 95% CI. **b.** Mean workspace area variations are depicted for the three groups across 12 training rounds. Error band = 95% CI.

As shown in Fig. 6a, all groups exhibited a significant increase in sitting workspace area after training (*p* < 0.001). The effect sizes (*partial η*^2^) were 0.90, 0.96, and 0.96 for the PR, AR, and VR groups, respectively. The increase was more pronounced in the AR and VR groups compared to the PR group, but was not significantly different between the AR and VR groups (*p* < 0.001 for AR vs. PR and VR vs. PR, *p* = 1.00 for AR vs. VR).

The mean workspace areas across 12 training rounds were depicted in Fig. 6b. For all groups, the workspace area increased sharply during the initial training rounds and stabilized by the end of training. Initially, all three groups started at a similar level, with error bands overlapping in the first training round. However, from the second round onward, the AR and VR groups consistently had a much larger workspace area than the PR group. The lower bounds of the error bands for AR and VR were higher than the upper bound of the PR group’s error band. Additionally, the error bands for AR and VR remained consistent with each other, showing similar fluctuations across the training rounds.

### C. Postural Control

Statistical analysis results of COP variables are summarized in Table I. After training, all groups exhibited a significant increase in COP total excursion during PSST. The increase was more pronounced in the AR and VR groups compared to the PR group, with no significant differences between the AR and VR groups. Only subjects in the PR group shifted their COP faster during PSST after training. The AR and VR groups showed no significant change in the mean COP velocity after training.

ApEn values revealed that all groups experienced significant improvements in postural control complexity and flexibility in coordinating body segments after training. The improvement was greater in the AR and VR groups compared to the PR group. There was no significant difference between the AR and VR groups.

**Fig. 7.**
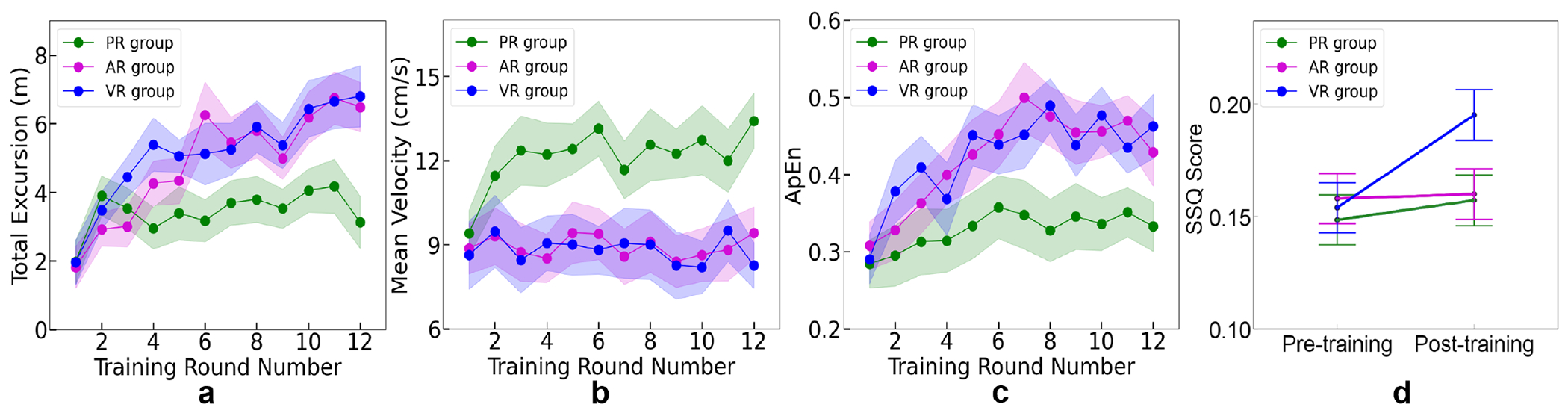
**a, b, c** depict the variations across 12 training rounds in total excursion, mean velocity, and ApEn, respectively. Error band = 95% CI. **d** Average SSQ scores for three groups in pre-training and post-training. Error bar = 95% CI.

The variations in COP variables across 12 training rounds were depicted in Fig. 7. In terms of total excursion, the PR group showed a sharp increase in the second round, followed by stabilization in subsequent rounds. However, both the VR and AR groups continued to increase throughout the training. Regarding mean COP velocity, only the PR group showed increased speed during training, while the AR and VR groups maintained their moving speed across 12 training rounds. Additionally, for the ApEn, the VR and AR groups showed a greater increment than the PR group in the initial rounds and stabilized at a higher level than the PR group by the end of training.

### D. Simulation Sickness Level

As shown in Table I and Fig. 7d, there was no significant change in the simulator sickness level for the PR and AR groups after training. Only the VR group exhibited a significant increase in the SSQ score after training (*F*(1, 60) = 26.34, *p* < 0.001, *partial η*^2^ = 0.31 ). Despite this increase, all subjects in the VR group reported that the discomfort was acceptable, and no severe simulator sickness symptoms were reported.

### E. Training Intensity

**Fig. 8.**
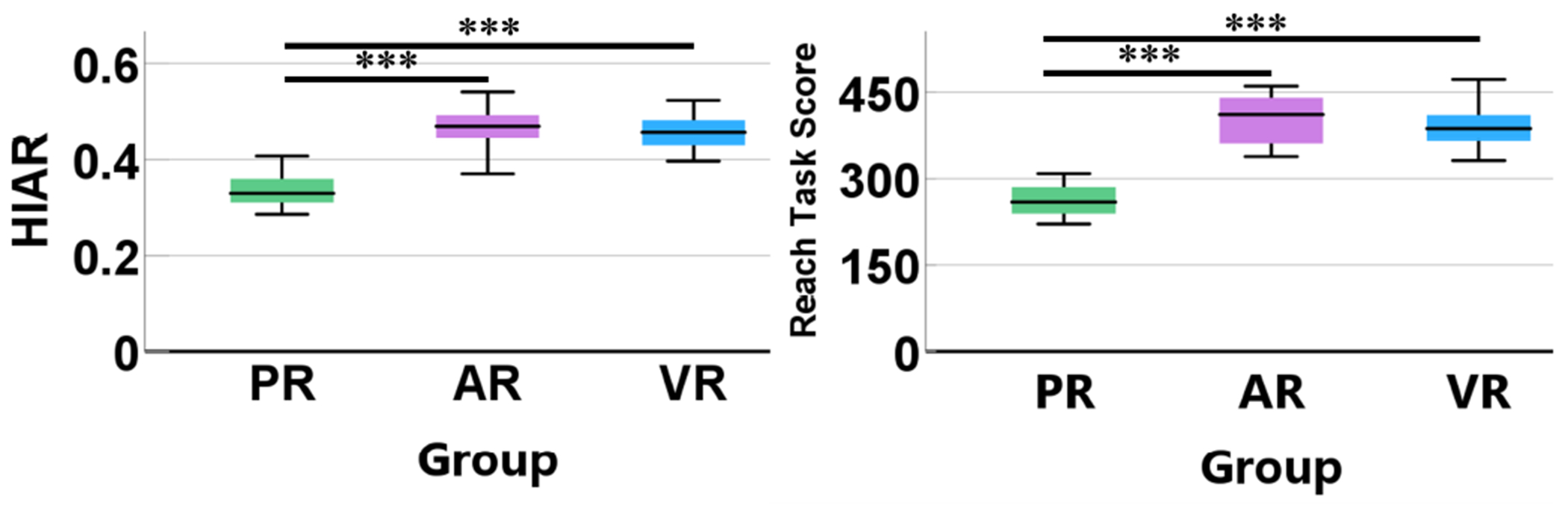
Box plots of HIAR and reach task score for the PR, AR, and VR groups. *** *p* < 0.001.

As shown in Fig. 8, subjects in the AR and VR groups underwent more intense training compared to the PR group. Both the HIAR and the reach task scores were significantly higher in the AR and VR groups than in the PR group (*p* < 0.001 for AR vs. PR and VR vs. PR). There were no significant differences between the AR and VR groups in terms of both the HIAR and the reach task score.

### F. Engagement and Enjoyment

**Fig. 9.**
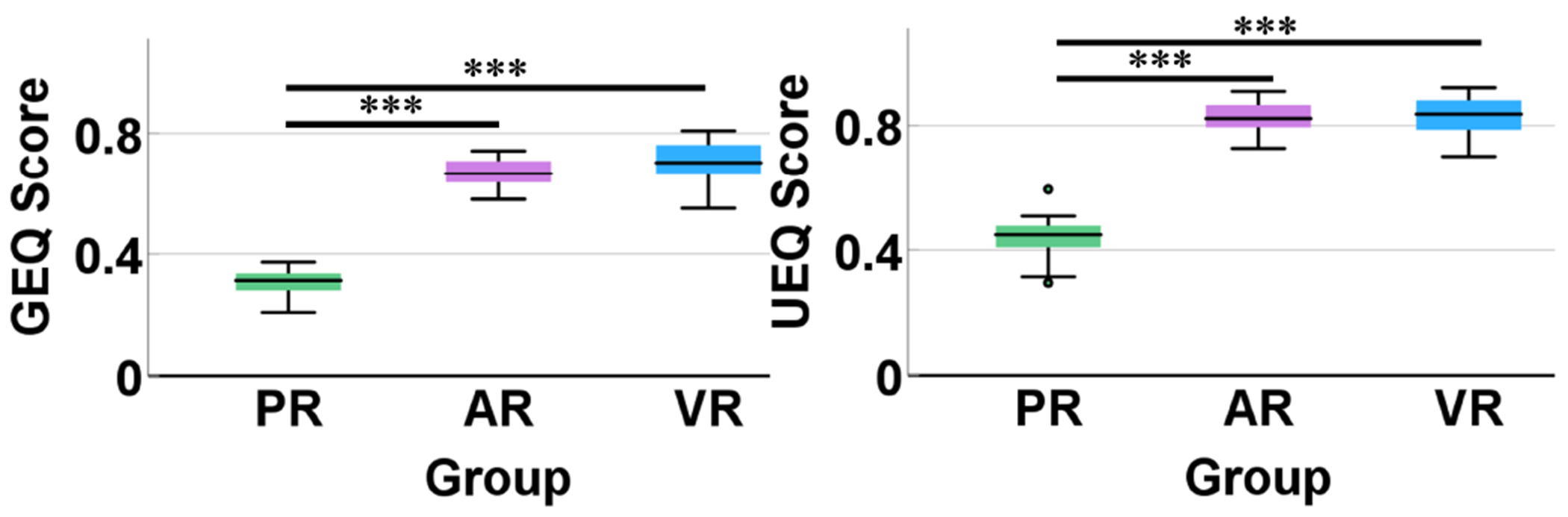
Box plots of GEQ score and UEQ score for the PR, AR, and VR groups. Outliers are denoted by little circles. *** *p* < 0.001.

As shown in Fig. 9, subjects in the AR and VR groups exhibited higher level of engagement and enjoyment when performing the training task, in contrast to the PR group. Both the GEQ score and the UEQ score were significantly higher in the AR and VR groups than in the PR group (*p* < 0.001 for AR vs. PR and VR vs. PR). No significant differences were observed between the AR and VR groups in terms of either the GEQ or UEQ scores.

## IV. Discussion

Our results show that introducing VR and AR into the TruST-assisted training protocol significantly improved training outcomes compared to TruST intervention alone, consistent with our first hypothesis. In addition, the VR group reported higher simulator sickness than the AR group after training, suggesting that AR is better suited for sitting posture training in conjunction with TruST intervention. This supports our second hypothesis.

### A. Effects of XR on Motor Performance

Functional reach movements require complex neuromuscular control to plan and achieve motor goals while maintaining posture equilibrium [2]. Previous research suggests that the XR interaction may induce various neurophysiological adaptations, such as improved interhemispheric balance, enhanced cortical connectivity, and increased muscle cortical representation [18]. Consequently, XR has the potential to stimulate neural plasticity, which positively impacts motor function. Key factors for promoting neural plasticity include adequate intervention time and repetitive goal-oriented practice [46].

In this study, the AR and VR groups performed the same number of reach movements as the PR group during training, but achieved higher HIAR and reach task score. This implies that subjects in the AR and VR groups spent more time beyond their stability limits, actively exploring and honing motor skills. In other words, they leveraged the TruST robotic platform more effectively–more frequently and longer use of the assistive force field during practice–which led to enhanced motor control ability in reaching the location of the most challenging targets. This might explain why the improvements in the FRTS and sitting workspace area are more pronounced in the AR and VR groups compared to the PR group. Future studies should explore hybrid protocols that combine XR with their robot-assisted posture training platforms. Such combinations may encourage subjects to proactively utilize the robotic assistive forces, potentially maximizing motor improvements.

### B. Effects of XR on Training Motivation

Previous studies suggest that the inherent gamification property of XR could enhance cognitive and emotional involvement, thereby encouraging voluntary participation during training [19], [46]. Our results align with the literature. Specifically, subjects in the AR and VR groups achieved significantly higher scores in GEQ and UEQ compared to the PR group, indicating greater engagement and enjoyment during seated postural training. For example, eighteen subjects in the AR group and nineteen subjects in the VR group reported a score of over four on the seven-point Likert scale for the following GEQ question: “Do you feel so engrossed in the game that you momentarily forgot you were undergoing a training task?” In contrast, no subject in the PR group reported a score above four on that question.

Previous studies have shown that subjects’ adherence to rehabilitation protocols and their active engagement while receiving the intervention importantly influence the treatment benefits [48], [49]. Thus, the attitude towards training plays a substantial role in the intervention effect. The psychological difference may explain why subjects in the AR and VR groups spent more of their training time in ‘intense’ reaching (i.e., higher HIAR) and achieved better training outcomes compared to the PR group. Our findings highlight the positive impact of gamification property of XR on posture training motivation. Future studies should consider integrating XR with gamified elements into their training protocols to enhance subject adherence and overall treatment effectiveness.

### C. Effects of XR on Postural Control

In this study, only the PR group exhibited rapid shifts in the upper body COM during training, resulting in significantly higher COP mean velocity in the post-training test. The AR and VR groups, on the other hand, maintained consistent COP moving speed across 12 training rounds but continued to increase the posture complexity during training. After training, the AR and VR groups showed significantly higher ApEn than the PR group. Our results suggest that the VR and AR groups employed different learning strategies compared to the PR group for the same reaching task. Specifically, the PR group prioritized faster movements, compromising posture stability for greater reach distance. The VR and AR groups focused on adjusting their postural configurations and fine-tuning body segment alignments to achieve extended reach.

Previous studies have highlighted two key parameters for assessing the learning process during task training: the learning slope at the beginning stage and the learning plateau near the end [50], [51]. In this study, the strategy employed by the VR and AR groups appears to be better suited for the reaching task. As shown in Fig. 6b, the VR and AR groups exhibited steeper slopes during the initial training rounds and reached higher plateaus at the end stage. This suggests that the VR and AR groups learned the task more rapidly initially and expanded their workspace larger than the PR group by the end of training. However, due to the limited training time with only one task, our findings in this study need further exploration. Future studies with extended training periods and diverse training tasks are necessary to better understand the effects of XR on the learning process during posture training.

### D. XR Game Application Design

Some studies employ open-source commercial games for XR posture training [6], [19]. However, excessive recreational and entertainment features within these games could cause cognitive overload and distract subjects from the rehabilitation goals [28]. In this study, we adopted suggestions from the literature [29], [46], [52] to develop a custom XR application that balanced entertainment with TruST-assisted postural training requirements. Our application involved randomized reach directions and clear functional reach targets with hierarchical difficulty levels. When the subject successfully reached a target, the virtual coin rotated to provide positive feedback. Besides, instead of traditional controllers, we opted for XR devices that allow direct hand interaction with virtual items, which facilitates the transfer of motor skills learned during training to activities of daily living. We will share our application with the research community following the IRB guidelines (access link: https://roar.me.columbia.edu/content/trust). Extending our approach to other studies may help develop novel rehabilitation-oriented XR applications suited for other robotic training platforms.

### E. Mitigate the Simulator Sickness

Motion sickness refers to the discomfort experienced after certain movements. Simulator sickness, a subtype of motion sickness, occurs in simulated environments due to mismatch between perceived visual motion and actual vestibular motion [36]. Prior studies highlighted the immersion level of XR systems as a critical factor affecting simulation sickness during training [28], [53]. Our results in this study align with the literature. Subjects in the PR and AR groups undertook training in the physical real environment, showing no significant change in SSQ scores after training. However, subjects in the VR group trained in a more immersive virtual environment, reported significantly higher SSQ scores after training. Our results suggest that the level of immersion can impact motion sickness, with higher immersion increasing the risk of simulator sickness. Therefore, AR appears better suited for sitting posture training when combined with TruST intervention. Future studies should consider using AR rather than VR when combined with RAT to mitigate simulator sickness.

Our findings highlight the positive relationship between immersion level and simulator sickness. We followed the methodology proposed by Selzer and Castro [47] to compute objective immersion scores for our AR and VR systems. Then we normalized the scores to the range 0–1 to position our systems on the RV continuum (Fig. 4). Although future studies may not employ the same XR devices and game applications as ours, researchers could still adopt this approach to measure immersion level and position their system on the RV continuum. If their XR device achieves a high immersion score (similar to our VR system), they should consider simplifying their XR training environments by limiting virtual item number to reduce the risk of simulator sickness.

Low refresh rate has been identified as another key factor contributing to simulator sickness in XR experiences [54]. Although the maximum refresh rate of our VR headset is 120 frame per second (fps), same as the gold standard [55], the actual tested refresh rate varied between 80 and 100 fps due to factors such as model size, model complexity, and virtual hand tracking load. Notably, our AR headset employs an optical see-through technique with transparent lenses, allowing for a direct view of the real world. Virtual objects are rendered onto the retina using low-power laser beams [56]. This innovative approach effectively bypasses the refresh rate issue and may explain the significantly lower SSQ score in the AR group compared to the VR group. Future studies should consider XR devices with optical see-through lenses or high refresh rate displays to mitigate simulator sickness.

### F. Study Limitations and Future Work

When integrating XR into RAT, it is crucial to consider not only potential benefits but also the safety risks arising from reduced awareness of reality. In this study, no subject in the AR group reported concerns about physical collisions with the TruST stationary frame. However, eighteen subjects in the VR group expressed worries about potential collisions with the robot frame during training. This psychological difference may account for the significantly lower FRTS improvements on the dominant and nondominant sides in the VR group compared to the AR group (see Fig. 5 D, ND). Our findings suggest that the presence of robotic platforms might hinder effective interaction with virtual items in the simulated environment. Future studies on combining XR with different robotic training platforms or exoskeletons should prioritize examining the potential implications of reduced reality perception. Researchers may consider using AR headsets rather than VR headsets or incorporating simulated virtual robot frames into the virtual environment to address this limitation.

Another limitation is that we only recruited healthy subjects in this study. Our XR-RAT combination approach has the potential to enhance seated postural control recovery in patients with neuromotor disorders and balance impairments. However, additional clinical testing is necessary as the XR-induced simulator sickness may be exacerbated in the patient population during training. In addition, our VR and AR systems heavily rely on intact visual information processing. Therefore, patients with visuomotor deficiencies may not benefit from our XR systems. To address these challenges, future work will explore novel XR techniques, including audio and haptic interactions, and directly evaluate the clinical applicability of our system with the patient population.

## V. Conclusion

In this study, we conducted a comparative study by integrating VR and AR into the TruST robotic platform for sitting posture training. Our results indicated that both VR and AR significantly enhanced the effectiveness of the postural control intervention delivered by TruST. The XR experience has the potential to enhance engagement and enjoyment during posture training. Consequently, combining VR or AR with RAT could increase training intensity, leading to improved motor performance and enhanced balance control. Notably, VR may introduce a higher level of simulator sickness compared to AR. Hence, AR may be more suitable than VR when combined with RAT. Overall, our findings help uncover the effects of introducing XR into RAT and provide insights for developing novel XR-enhanced RAT platforms. Future studies should investigate the neuromuscular control mechanisms underlying the XR-introduced motor changes and the clinical potential of XR-enhanced RAT in patients with neuromotor disorders.

## Figures and Tables

**TABLE I:** Baseline (T0) and Post-Training (T1) Assessment Results of COP Variables and Simulator Sickness Level

	PR group (a)			AR group (b)			VR group (c)			*Pab*	*Pac*	*Pbc*

	T0	T1	*p*	T0	T1	*p*	T0	T1	*p*			
Total excursion (m)	1.91±0.18	3.26±0.24	***	1.85±0.17	4.58±0.36	***	1.89±0.20	4.52±0.29	***	***	***	1.00
Mean velocity (cm/s)	9.01±0.80	11.97±0.62	***	8.76±0.55	8.69±0.89	0.75	8.84±0.66	8.92±0.67	0.71	***	***	0.90
ApEn	0.30±0.02	0.36±0.04	***	0.31±0.02	0.40±0.04	***	0.30±0.02	0.42±0.04	***	***	***	1.00

SSQ score	0.15±0.03	0.16±0.03	0.28	0.16±0.02	0.16±0.03	0.81	0.15±0.02	0.20±0.02	***	0.81	***	0.02*

1 Values are presented as mean ± standard deviation.

2 *p* values for comparisons within and between groups are displayed.

* *p* < 0.05,

** *p* < 0.01,

*** *p* < 0.001.

3 *p_ij_* denotes the *p*-value between group i and group j.
